# Botanical Description of British Plants

**Published:** 1807-08

**Authors:** 


### *Botanical Description of British Plants.*

[ Continued from Vol. xvii. page 558—566. ]

19. Prunella. *P. vulgaris. Brunella vulgaris. B. major fol. non dissecto.*

*Ang.* Common Self-heal.

*Gen. Desc.* Filaments forked, one of the divisions bearing anthers. Summit cloven.

*Spec. Desc. Leaves* egg-oblong, serrated, on leaf-st., opposite. *Cal.* up. lip lopped, three-toothed, seven ribbed. *Bloss.* blue, purple, or white; *up. lip* slightly notched; *lower* mid. segm. jagged. *Sum.* segm. rolled back. *Stem.* in sunny situations trailing, a finger long; in woods upright, a foot high; thinly hairy. *Meadows, pastures.* Bloss. August.

*Use.* It is *astringent* and *vulnary,* but it is rarely used at present, except by country people; by them it is bruised and applied to fresh wounds; they take it also in broths and apozems for *spitting of blood;* and, in *bloody flux* and other *hæmorrhages,* they use it by way of injection.—*Lightfoot.*

Didynamia. Angiospermia.

20. Euphrasia. *E. officinalis.*

*Ang.* Eyebright.

*Gen.*

*Gen. Desc.* Cal. four-cleft, cylindrical. Anthers, the lower ones with one thorny to be at the base. Caps. two-celled, egg-oblong. Seeds few, leaning backwards.

*Spec. Desc.* *Leaves* egg-shaped, streaked, sharply toothed, sitting, mostly opposite. *Bloss.* bluish white; upper-lip, cloven, segm. three-toothed, streaked with purple; lower-lip, segm. cloven; mid. segm. with a yellow blotch; lat. seg. with three purple lines. *Heaths, dry grounds,* Bl. July, September.

*Use.* It is a weak *astringent*; it was formerly in repute as a remedy for impaired vision; especially in eyes weakened by long exertion, and those that are dim and watery, as in a senile state; strong testimonies are offered of its efficacy at very advanced ages; but not one in modern practice. The usual mode of external application was the juice mixed with wine and a little honey. It is constantly used by the Icelanders; and is recommended also in the *jaundice.*—*Woodville.* It will not grow but when surrounded by plants taller than itself.—Cows, horses, sheep, and goats eat it; swine refuse it.—*Withering.*

21. Pedicularis. *P. palustris.*

*Ang.* Marsh lousewort.

*Gen. Desc.* Cal. five-cleft. Caps. two-celled, sharp pointed, slanting. Seeds few, coated.

*Spec. Desc.* *Stem.* branched, 1 foot high, angular, purplish. *Leaves* winged, leafits wing-cleft. *Flowers* solitary, axillary, purple, sometimes white; helmet a little tooth on each side; lip slanting. Cal. two-lipped, crested with callous dots. *Marshes, &c.* Bl. June, July.

*Use.* This is a very unwelcome guest in meadows, being very disagreeable to cattle. Goats eat it; swine are not fond of it; horses, cows and sheep refuse it.—*Withering.*

22. Pedicularis. *P. sylvatica.*

*Ang.* Common lousewort.

*Gen. Desc.* As above.

*Spec. Desc.* *Stem.* branched, very short. Cal. oblong, angular, smooth, green within; purplish without, segm. toothed. *Bloss.* lip neat-shaped, purple, slenderer than cal. tube compressed. *Branches* trailing. *Wet pastures, heaths.* Bl. June, July.

*Use.* The expressed juice, or a decoction of this plant has been used with advantage as an injection for *sinuous ulcers.*—It is said, that if the healthiest flock of sheep be fed with it they become scabby and scurfy in a short time; time; the wool will get loose, and they will be over-run with vermin.—Cows and swine refuse it.—*Withering.*

23. ANTIRRHINUM. *A. Elatine.*

*Ang.* Sharp-pointed Fluellin, or snap-dragon.

*Gen. Desc.* Cal. five-div. Bloss. bulging at the base, or ending in a spur. Caps. two-celled, many-seeded, opening at the top, and the divisions bent back.

*Spec. Desc.* *Leaves* halberd-shaped, alternate; lower ones opposite. *Fruit-st.* white, in flower expanding, then declining. *Stem.* trailing. *Corn-fields.* BL. Aug. Oct.

*Use.* This is considerably more bitter than the other species of it; and it is said to have been used successfully in cases of *foul ulcers,* and in *cutaneous eruptions.*

24. ANTIRRHINUM. *A. linaria.*

*Ang.* Yellow toad-flax. Snap-dragon. Butter and eggs.

*Gen. Desc.* *As above.*

*Spec. Desc.* *Leaves* spear-strap-shaped, crowded, alternate. *Stem.* upright, smooth *spikes* terminating, sitting. *Flowers* tiled. *Bloss.* petals woolley, orange; *lower-lip,* segm. circular, mid. segm. much smaller, projecting part orange, woolly; the rest pale yellow, smooth. *Nect.* long, awl-shaped. *Barren fields, road sides.* BL. July, Sept.

*Note.* *There are four varieties, for which, particularly Pesoria, see Bot. Arr. l. c.*

*Use.* An ointment prepared from the leaves, and invented by Dr. Wolph, Physician to the Landgrave of Hesse, has been used in the *piles,* and it is said to afford relief.—*Light.*—*With.* The leaves in infusion are *diuretic* and *purgative,* and are recommended in *dropsies.* It has also been used in *jaundice.* An infusion of the flowers is said to be very efficacious in cutaneous disorders.—*Woodville.* The expressed juice mixed with milk is a poison to flies, and the smell of the flowers has the same effect on them. Cows, horses and swine refuse it. Sheep and goats are not fond of it.—*Withering.*

25. SCROPHULARIA. *S. nodosa.*

*Ang.* Great fig-wort. Kernel-wort. Knobby-rooted fig-wort.

*Gen. Desc.* Cal. five-cleft. Bloss. five-div. tube globula; lower segment reflected. Caps. two-celled, partition double.

*Spec. Desc.* *Leaves* oblong, heart-shaped, three-fibred at the base. Stem sharp-cornered. *Cal.* toothed and membraneous. *Bloss.* tube filled with honey; *upper segm.* purple, the rest pale green: *two lat.* expanding, *lower* rolled back. *Woods, moist hedges.* BL. July.

*Use.* This plant is hardly known in modern practice; but its rank smell, and the bitter taste of the leaves seem to indicate some active properties.—Swine infected with the *scab*, have been cured by being washed with a decoction of the leaves.—Wasps greatly resort to the flowers.—Goats eat it; cows, horses, sheep and swine refuse it.—*Withering*.

26. DIGITALIS. *D. purpurea. Virgia regia major.*
*Ang.* Fox-glove.

*Gen. Desc.* Cal. five-div. Bloss. bell-shaped, five-cleft, bellying. Caps. egg-shaped, two-celled, many-seeded.

*Spec. Desc.* *Cal.* segm. egg-shaped, acute. *Bloss.* blunt, up.-lip. nearly entire; purple, mottled within; inversely conical, swelling on the innerside; the bellying part with spots like little eyes. *Leaves* sitting, wrinkled, scalloped, teeth small, deep; a net-work of fleshy veins underneath, a little woolly. *Flowers* in long terminating spikes, all pointing one way. *Hedge banks, hills, in dry soil, but rarely in flat ground.* BL. June, July.

*Use.* The leaves, which have a nauseous bitter taste, have long been used externally to *sores* and *scrophulous tumours* with considerable advantage. Internally this plant has been reckoned efficacious in *epilepsy, scrophula* and *phthisis*; but from want of caution it was found a dangerous remedy. It was discovered by Dr. Withering to be a powerful diuretic, and has been successfully used by him, and by succeeding practitioners in the cure of the dropsy; in which, under certain precautions, it is a valuable and important medicine. *See Withering's Acc. of the Fox-glove pub.* 1785. But, administered with less attention or less skill, it was proved either useless or deleterious. *Mem. Med. Soc. vol. ii.* It has a very remarkable power of lowering the pulse.—*Woodville.* It is certainly a very active medicine, and seems lately to have attracted more attention than modern practice was heretofore accustomed to bestow on it. (*See Beddoes on Pulmonary Consumption. Ferriar on Digitalis, &c.* Six or seven spoonfuls of the decoction of the leaves are violently *emetic* and *cathartic*. It is said to be useful in *scrophulous* cases taken inwardly for some time; and the bruised leaves, or an ointment made of them, applied outwardly.—*Lightfoot.*

### CLASS. XV. TETRADYNAMIA.

### Tetradynamia. Siliculosa.

1. MOENCHIA. *M. sativa. Myogrum sativum.*
*Ang.* Gold of pleasure. Common camline.

*Gen*

*Gen. Desc.* Pouch entire, egg-shaped, crowned with the style, valves rather convex, parallel to the partition; cells with many seeds.

*Spec. Desc.* *Pouches* inversely egg-shaped, on long fruit-st. each side bellying out, but with a cavity pressed inwards, which disappears by cultivation. *Stem.* angular at top, slightly hairy, cloathed with leaves. *Leaves* alternate, spear-shaped, (arrow-sh. at base) half embracing. Stem, slightly toothed, hairy. *Bloss.* yellow.—*Corn and flax-fields.* Bl. May, June.

*Use.* This plant is an object of cultivation in Germany, for the sake of an expressed oil from the seeds, which is used by the inhabitants for medicinal, culinary and œconomical purposes. The seeds are a favourite food with geese. Horses, cows, goats and sheep eat it.—*Withering.*

2. Crambe. *C. maritima.*

*Ang.* Sea colewort. Cliff kale.

*Gen. Desc.* The four longer filaments cloven at top, one of the clefts bearing the anther. Seed-vessel simple, globular, deciduous.

*Spec. Desc.* *Stems* many, spreading, branched, smooth, *Root-leaves* on leaf-st. v. large, spreading on the ground, waved, jagged and indented and curled, smooth, tea-green, tinged with purple. *Stem-l.* sitting, fleshy, glaucous, indented and curled, smooth. *Pouch* at first egg-shaped, afterwards nearly globular. *Bloss.* large white, claws often purple both of petals and filaments. *Sandy sea shores.* Bl. May, June.

*Use.* The young and tender leaves are boiled as cabbage, or when first shooting are blanched and eaten as asparagus; but when full grown, they occasion giddiness. Horses, cows, goats and swine eat it.—*Withering.*

3. Isatis. *I. tinctoria.*

*Ang.* Wildwood.

*Gen. Desc.* Pouch simple, oval spear-shaped, compressed, one-celled, one-seeded, crowned by the style.

*Spec. Desc.* *Pouches* oblong, pendant, on slender fruit-st. chesnut-colour. *Root-leaves* scalloped, *stem-l.* arrow-shaped. *Stem* branched, woody. *Calyx* yellow. *Petals* yellow, notched at the end. *Corn-fields, rather rare.* Bl. June, July.

*Use.* This plant is much used by dyers for its *blue* colour; and it is the basis of many other colours; it is cultivated for their use. With the juice of this plant, it is said that the ancient Britons painted their bodies, to render themselves more terrible to their enemies. Cows eat it; horses, sheep and goats refuse it.—*Withering.*

4. Lepidium. *L. latifolium.*

*Ang.* Dittander pepper-wort. Poorman's pepper. Common dittander.

*Gen. Desc.* Pouch notched, compressed; valves sharply keeled. Cells one-seeded.

*Spec. Desc.* *Leaves* egg-spear-shaped, entire, serrated. *Stem* branched, zigzag. *Flowers* numerous, in panicles, white.—*Meadows and pastures.* Bl. June, July.

*Use.* This is one of the acrid *antiscorbutics*; and was formerly used in the place of *Horse-raddish.* An infusion of it is emetic. *Withering.*

5. Thlaspi. *T. arvense.*

*Ang.* Treacle mustard. Penny cress.

*Gen. Desc.* Pouch inversely heart-shaped, notched; valves like a keeled boat, often winged with a border; cells many-seeded.

*Spec. Desc.* *Pouches* round, flat. *Leaves* oblong, toothed, smooth, arrow-shaped at the base, embracing the stem. *Stem* 2 feet high, with seven or eight membranaceous edges. *Bloss.* white. *Corn fields, muddy soil.* Bl. June, July.

*Use.* The seeds of this plant have the acrimony of mustard; and the whole has something of a garlick flavor. When it is eaten by cows, it imparts a disagreeable taste to the milk.—Cows, goats, and swine eat it; horses and sheep refuse it.—*Withering.*

6. Thlaspi. *T. bursa pastoris.*

*Ang.* Shepherd's purse. Shepherd's pouch.

*Gen. Desc.* *As above.*

*Spec. Desc.* *Pouches* compressed, triangularly inversely heart-shaped, smooth, without a border. *Root-leaves* wing-cleft, sometimes entire. *Stem-leaves* entire, strap-spear-shaped, embracing the stem, fringed. *Bunches* long, flatted, terminating. *Cal.* hairy. *Petals* entire, white. *Anthers* woolly. *Summit* circular, fringed, concave. *Stem* 2 inches to 3 feet high. *Rubbish, road sides, walls, &c.* Bl. March, Sept.

*Use.* It is insipid to the taste; but it is recommended by many writers in *hæmorrhages* of all kinds, both as an internal or external application, either in man or beast.—*Lightfoot.*

( To be continued. )

